# Diabetic Ketoacidosis Is Associated with Lower Serum Sphingolipids but Higher β-Hydroxybutyrate and Lactate: A Pilot Study

**DOI:** 10.3390/pathophysiology32030029

**Published:** 2025-06-26

**Authors:** Ibrahim Aslan, Tuğçe Çeker, Tayfun Ustabaş, Vuslat Zorlu, Çağatay Yılmaz, Mutay Aslan

**Affiliations:** 1 Endocrinology Clinic, Antalya Research and Education Hospital, The University of Health Sciences, Antalya 07000, Turkey; ibrahimaslansbu@gmail.com; 2 Department of Medical Biochemistry, Akdeniz University Medical Faculty, Antalya 07000, Turkey; tugceker159@gmail.com (T.Ç.); ccagatayyilmaz@gmail.com (Ç.Y.); 3 Internal Medicine Clinic, Antalya Research and Education Hospital, The University of Health Sciences, Antalya 07000, Turkey; tayfunustabas1@gmail.com (T.U.); vusltzorlu@gmail.com (V.Z.)

**Keywords:** diabetic ketoacidosis, sphingolipids, neutral sphingomyelinase, β-hydroxybutyrate, tumor necrosis factor alpha

## Abstract

**Background/Objectives**: Diabetic ketoacidosis (DKA) is an acute and severe complication of diabetes mellitus, marked by hyperglycemia, ketosis, and acidosis. It is associated with significant metabolic and inflammatory adjustments that can impact multiple biochemical pathways. This study aimed to determine the serum sphingolipid profile in DKA and investigate its relationship with neutral sphingomyelinase (N-SMase), pro-inflammatory cytokines, β-hydroxybutyrate (β-OHB), and lactate levels. **Methods**: Thirty-three participants were divided into three groups: control (BMI ≤ 30, no health issues), obese (BMI > 30), and DKA (BMI ≤ 30). Sphingomyelins (16:0–24:0 SMs) and ceramides (C16–C24 CERs) were measured using ultra-fast liquid chromatography combined with tandem mass spectrometry (LC-MS/MS). N-SMase, interleukin 1 beta (IL-1β), and tumor necrosis factor alpha (TNF-α) levels were assessed by enzyme-linked immunosorbent assay. Evaluations were done in the DKA group before and after standard clinical treatment for DKA (post-DKA group), which included intravenous insulin therapy, fluid resuscitation, and electrolyte replacement, as per established clinical guidelines. **Results**: β-OHB levels were significantly higher in the DKA group than in the control, obese, and post-DKA groups. Although β-OHB levels decreased in the post-DKA group, they remained elevated compared to the control and obese groups. Lactate levels were also higher in the DKA group, with a significant decrease in the post-DKA group. TNF-α and IL-1β were higher in the obese group compared to control and DKA groups, and TNF-α decreased significantly in the post-DKA group compared to DKA. N-SMase, 16:0–18:0 SMs, and C18-C24 CER levels were lower in the DKA and post-DKA groups compared to obese and control groups. Serum β-OHB and lactate levels were significantly correlated with S1P, total CER, total SM, and N-SMase values. **Conclusions**: The study reveals significant metabolic and inflammatory differences in DKA and post-DKA states, suggesting a relationship between sphingolipids, N-SMase, and these alterations, which could offer insights into DKA pathophysiology and therapeutic targets.

## 1. Introduction

Sphingolipids serve as key components in the structure of cell membranes, and recent research has highlighted their role in the generation of various lipid mediators [1]. At the core of sphingolipid metabolism is ceramide. Ceramide is synthesized through the interaction of serine and palmitoyl CoA. Additionally, ceramide can be produced through the action of the enzyme sphingomyelinase, which hydrolyzes sphingomyelin [2]. After its formation, ceramide is either converted to ceramide-1-phosphate by ceramide kinase or utilized in the synthesis of sphingomyelin and glycosphingolipids [2]. Ceramide may also undergo breakdown into sphingosine via the action of ceramidase enzymes. Sphingosine, in turn, can be phosphorylated by sphingosine kinases to form sphingosine-1-phosphate. This phosphorylated metabolite, sphingosine-1-phosphate, acts as a potent bioactive mediator and has recently been implicated in the inflammatory response [3].

Plasma ceramide levels are elevated in individuals with severe obesity [4]. Various mechanisms have been identified that explain how ceramides contribute to the development of insulin resistance, particularly at the receptor and post-receptor stages of insulin action. One of the key mechanisms involves the inhibition of glucose transporter type 4 (GLUT-4) translocation by ceramides, which impairs glucose uptake in insulin-sensitive tissues, thus leading to insulin resistance [5]. Furthermore, elevated ceramide levels are known to interfere with insulin’s ability to activate protein kinase B, further promoting insulin resistance [6].

Obesity can cause chronic inflammatory condition. In obese patients, increased adipose tissue also functions as an active endocrine organ by secreting proinflammatory cytokines such as interleukin 6 (IL-6), IL-1-beta, TNF alpha [7]. Chronic inflammation in obese patients causes lipolysis, thereby increasing fatty acid release from peripheral tissues [8]. While the resulting fatty acids are used in ceramide formation, pro-inflammatory cytokines induce ceramide formation through enzymatic and receptor pathways [9]. Ceramides are also known to increase the formation of pro-inflammatory cytokines in obesity, a precondition that can contribute to decreased insulin sensitivity [10]. Pro-inflammatory cytokines also induce recycling pathways in which ceramides are deactivated to form sphingosine [9].

Diabetic ketoacidosis is an acute and life-threatening complication of diabetes. The clinical definition of diabetic ketoacidosis can be summarized as an acute condition of uncontrolled diabetes requiring immediate insulin and intravenous fluid therapy [11]. Diabetic ketoacidosis is usually seen in patients with type 1 diabetes, but DKA can also develop in patients with type 2 diabetes. DKA can be seen in patients with type 2 diabetes in cases of severe infection, trauma, cardiovascular disease [11]. The prevalence of diabetic ketoacidosis is reported to be 3–8/1000. Diabetic ketoacidosis presents a clinical picture with hyperglycemia, ketosis and acidosis [12].

In insulin deficiency, glycogenolysis occurs in the liver, proteolysis takes place in muscle and lipolysis arises in adipose tissue. The secretion of glucose from the liver, the release of amino acids and fatty acids from muscle and adipose tissues increases [12]. Increased amino acids are used in liver gluconeogenesis while glucose uptake of peripheral tissues is also reduced due to insulin deficiency and/or resistance which also triggers hyperglycemia [11,12]. Acetyl-CoA, which is released in excess due to the oxidation of fatty acids in the liver, causes an increase in ketone production also termed ketogeneses. Beta-hydroxybutyrate constitutes 70–85% of the ketoacids produced in the liver and released into the circulation, while acetoacetate comprises 20% and acetone represents 2%. Blood ketone values are important in the evaluation of ketoacidosis [11,12].

Once considered as “metabolic waste”, ketones have become a major focus in cardiometabolic research [13]. Recent discoveries have revealed that ketones, such as acetoacetate and its precursor beta-hydroxybutyrate, are signaling molecules that elicit anti-inflammatory effects [14]. Ketogenic diet (KD) causes a decrease in TNF-alpha, IL-1-alpha, IL-1-beta, and IL-6 levels [15], while lowering ceramide levels in humans [16]. Beta-hydroxybutyrate also causes a decrease in ceramides in rat muscle cells [17].

In this context, the hypothesis of this work was that increased beta-hydroxy butyrate levels in DKA could cause quantitative variations in circulating levels of ceramides and pro-inflammatory cytokines. In the present study, beta-hydroxybutyrate levels, serum sphingolipids and proinflammatory cytokines were investigated in patients with DKA. The results were compared with those of obese and healthy individuals.

## 2. Materials and Methods

### 2.1. Study Population

This study included 33 volunteers, who applied to the Endocrinology Clinic at Antalya Research and Education Hospital. All cases of ketoacidosis had previously been diagnosed with type I diabetes. All DKA patients were using different doses of insulin prior to enrolment in the study. In the first stage of the study, 3 groups were formed. The control group, mean age of 30 ± 3.72, included 11 female and 1 male participants *(n* = 12) without underlying health issues and a BMI of 30 or less. The obese group, mean age of 30.18 ± 8.73, included 9 female and 2 male participants (*n* = 11) with a BMI above 30. The DKA group, mean age of 35.50 ± 17.01, included 5 female and 5 male participants (*n* = 10) all of whom had a body mass index (BMI) of 30 or less. Individuals were excluded if they had taken fibrates, niacin, or omega-3 supplements within the last three months, had a history of psychosis or cancer, were pregnant, or had active liver disease, kidney failure, or unstable angina. The study received ethical approval from the Clinical Research Ethics Committee of Akdeniz University (Reference no: KAEK-752 Date: 27 September 2023).

### 2.2. Laboratory Measurements

For control and obese groups, blood samples were collected once. For the DKA group, blood samples were collected twice: at the time of admission (DKA) and before oral feeding following recovery from ketoacidosis (post-DKA). The duration of ketoacidosis was between 4–24 h in patients diagnosed with DKA and during this period, they did not enter hypoglycemia. All DKA patients were administered 15–20 mL/kg (1–1.5 L), 0.9% normal saline over the first hour after hospitalization to avoid dehydration. Blood pH and HCO_3_ levels were measured by blood gas analyzer (Radiometer ABL800, Kopenhag, Denmark). C-reactive protein (CRP), glucose, blood urea nitrogen (BUN), creatinine, lactate, Na^+^, K^+^, Cl^−^, Ca^2+^, total cholesterol, high-density lipoprotein cholesterol (HDL-C), triglyceride, albumin, alanine aminotransferase (ALT), aspartate aminotransferase (AST) and lipase were measured by a clinical chemistry analyzer (Beckman Coulter AU5800, Brea, CA, USA). Thyroid stimulating hormone (TSH), free thyroxine (fT4) and insulin were measured by an immunoassay system analyzer (Beckman Coulter DxI 800, Brea, CA, USA). Urine ketone was measured by an automated urine analyzer (DIRUI FUS-200, Changchun, China) while HbA1c was measured by an automated glycohemoglobin analyzer (Tosoh HLC-723G11, Tessenderlo, Belgium).

### 2.3. Measurement of Sphingomyelins and Ceramides

SMs and CERs were quantified using a multiple reaction monitoring (MRM) technique, optimized for accuracy. Ultra-fast liquid chromatography (UFLC, LC-20 AD UFLC XR, Shimadzu Corporation, Japan) in conjunction with tandem mass spectrometry (LC-MS/MS-8040, Shimadzu Corporation, Kyoto, Japan) was used to perform the analysis in accordance with the procedures described in earlier studies [4]. SMs and CERs standards were obtained from Avanti Polar Lipids (Alabaster, AL, USA) and included sphingosine-1-phosphate (S1P), C16 SM, C18 SM, C24 SM, C16 CER, C18 CER, C20 CER, C22 CER, and C24 CER. The internal standard used, C16 CER d18:1/16:0 (Palmitoyl-U-13C16), was supplied by Cambridge Isotope Labs (Andover, MA, USA). All standard solutions were dissolved in methanol at 40 °C with sonication. A C18 HPLC column (XTerra, 2.1 mm × 50 mm, Waters, Milford, MA, USA) was used for chromatographic separation, which was carried out at 60 °C and 0.45 mL/min. The gradient elution used solvent-A (water-acetonitrile-isopropanol, 8:1:1 *v*/*v*/*v* addition to 10 mM ammonium formate) and solvent-B (acetonitrile-isopropanol, 9:1 *v*/*v*). The flux began with 65% solvent-B (0–2 min), increased to 90% (2.01–13 min), peaked at 100% (13.01–20 min), and returned to 65% (20.1–23 min). Positive electrospray ionization (ESI) mode was the system’s mode of operation.

The precursor and product ion pairs for each sphingomyelin and ceramide were as follows: S1P, m/z 380.10 → 264.40; C16 SM, m/z 703.30 → 184.20; C18 SM, m/z 731.40 → 184.20; C24 SM, m/z 815.50 → 184.20; C16 CER, m/z 538.50 → 264.40; internal standard C16 CER*IS, m/z 554.30 → 264.30; C18 CER, m/z 566.30 → 264.40; C20 CER, m/z 594. 60 → 264.50; C22 CER, m/z 622.60 → 264.40; and C24 CER, m/z 650.40 → 264.30. A calibration range of 39 to 625 ng/mL was applied for quantification and the total analysis time per sample was 23 min. Supplementary Table S1 lists the LC-MS/MS validation parameters for every sphingolipid that was tested. Assay repeatability (intra-day CV), reproducibility (inter-day CV), and accuracy were determined for all standards used. For intra-day coefficient variation (CV) standards were analysed repeatedly the same day. For inter-day CV standards were analysed repeatedly over 2 days. The results demonstrate and acceptable accuracy range between 85–115% for most standards. The intra-day and inter-day CVs are ≤15% and meet standard guidelines.

### 2.4. Serum Sample Preparation for Mass Spectrometry

Sphingolipids were extracted from serum using established protocols [4]. First, 500 µL of serum that had been diluted 1:20 (*v*/*v*) with distilled water was mixed with 2 µL of internal standard solution (5 μg/mL). The mixture was vortexed, followed by the addition of 375 µL of chloroform/methanol (1:2, *v*/*v*). After sonication for 30 s, 100 µL of water was added, and the samples were vortexed for 5 min. Before centrifuging the mixtures at 2000 g for five minutes to separate the supernatant, they were allowed to stand at room temperature for thirty minutes. To further enhance phase separation, 125 µL each of chloroform and water were added, and the samples were vortexed again. The organic phase was then transferred to glass tubes and allowed to dry at room temperature with a steady nitrogen flow using a height-adjustable gas distribution device (VLM, Bielefeld, Germany). After dissolving the dried residue in 100 µL of methanol, 10 µL of injection volume was taken into the LC-MS/MS for measurement.

### 2.5. Measurement of Serum β-Hydroxybutyrate Levels

Serum β-hydroxybutyrate levels were measured using a colorimetric assay kit (Cayman Chem., Ann Arbor, MI, USA, Catalogue number: 700190). This test requires 3-hydroxybutyrate dehydrogenase’s enzymatic conversion of D-3-hydroxybutyrate to acetoacetate and NAD+ to NADH. In the presence of diaphorase, NADH reacts with WST-1 to form a colored formazan product, which is measured spectrophotometrically at 450 nm to directly determine β-hydroxybutyrate concentrations. The concentrations were calculated in mM based on a standard curve constructed with known amounts of β-hydroxybutyrate standards. The absorbance value of 0 mM standard was subtracted from itself and all other standards and samples to obtain the corrected absorbance. β-hydroxybutyrate levels were calculated from the formula below:

β-OHB (mM)= [Corrected absorbance − (y intercept)/Slope] × Dilution

### 2.6. Measurement of Serum N-SMase, IL-1β, and TNF-α Levels

Levels of N-SMase, IL-1β, and TNF-α in serum were measured using enzyme-linked immunosorbent assay (ELISA) kits (BT LAB, Hangzhou, China; Cat. No. E6866Hu, E0143Hu, E0082Hu, respectively). The assays were carried out in compliance with the manufacturer’s guidelines. Serum samples were added to ELISA plates pre-coated with monoclonal antibodies (mAb) for N-SMase, IL-1β, and TNF-α. After incubation with biotinylated monoclonal antibodies, streptavidin-HRP was added. Lastly, a colored reaction was produced using tetramethylbenzidine (TMB) as a substrate, and the reaction was monitored at 450 nm using a microplate reader. N-SMase, IL-1β, and TNF-α concentrations were determined by sample absorbance values via a standard curve constructed from established reference standards.

### 2.7. Statistical Analysis

Statistical analyses were performed using GraphPad Prism (version 9.0.0, GraphPad Software, Inc., La Jolla, CA, USA) and Sigma Stat software (version 2.03, Systat Software, San Jose, CA, USA). The chosen statistical methods ensure appropriate analysis based on the data distribution and study design, minimizing the risk of erroneous conclusions while maximizing statistical power. In the first stage of the study, we formed three groups: the control group (*n* = 12), obese group (*n* = 11), and DKA group (*n* = 10). Blood samples were collected once for the control and obese groups, whereas for the DKA group, samples were collected twice—at admission and before oral feeding following recovery from ketoacidosis. This resulted in a total of four groups for statistical analysis. To determine the appropriate statistical tests, we first conducted a normality test. If the data followed a normal distribution, parametric tests were applied; otherwise, nonparametric tests were used. A *p*-value of less than 0.05 was considered statistically significant. The specific statistical tests used for each measurement are detailed in the figure and table legends.

We have performed two separate ANOVA analysis for the comparison of the four groups in which DKA and post DKA groups were included in the ANOVA analysis separately. 1. Parametric analysis (if normality was met): One-way ANOVA, followed by Tukey’s multiple comparison test to determine differences between groups. 2. Nonparametric analysis (if normality was not met): Kruskal-Wallis one-way analysis of variance, followed by Dunn’s multiple comparison test for pairwise group comparisons.

Comparison of DKA and post-DKA groups (paired samples): 1. Paired *t*-test (if normality was met). 2. Wilcoxon matched pairs signed rank test (if normality was not met).

Comparison of control and obese groups (for insulin and HOMA-IR values measured only in control and obese groups): Unpaired *t*-test, as only two independent groups were compared.

Correlation coefficients and scatter plots were used to examine the relationships between laboratory values and other biomarkers. Linear regression analysis was applied to assess the extent and nature of these relationships. By using these statistical methods, the study not only identified significant relationships between biomarkers but also assessed how well specific variables predicted others, enhancing the biological interpretation of the data. The reported R^2^ values in the correlations, provides a quantitative measure of how well the measured parameters are related. Specifically, for the linear regression analyses, the R^2^ value indicates how much of the variation in the dependent variable such as S1P, total CER, N-SMase, can be explained by the independent variables such as β-hydroxybutyrate or lactate. As an example, R^2^ of 0.67 means that 67% of the variability in the dependent variable (*y*-axis) can be accounted for by changes in the independent variable (*x*-axis), while the remaining 33% of the variability in y is due to other factors or random variation not explained by x, suggesting a strong relationship between the two. On the other hand, an R^2^ of 0.2 would indicate a weaker relationship, where only 20% of the variation in the dependent variable is explained by the independent variable. Statistical significance was established with *p*-values less than 0.01 for β-hydroxybutyrate and lactate and less than 0.05 for other analyses.

Post hoc power calculations were carried out using the G Power software version 3.1.9.7. (University of Duesseldorf, Düsseldorf, Germany). As stated above, in the first stage of the study, we established three groups: the control group (*n* = 12), obese group (*n* = 11), and DKA group (*n* = 10). Blood samples were collected once for the control and obese groups, whereas for the DKA group, samples were collected twice—at admission (DKA) and before oral feeding following recovery from ketoacidosis (post-DKA). This resulted in a total sample size of 43 for one-way ANOVA and linear regression analysis, while the total sample size was 20 for paired *t*-tests (DKA/post-DKA groups). The effect size (ES) was determined by Cohen’s value, which is interpreted as follows, 0.2 to 0.3 for small effects (a subtle difference between the groups); roughly 0.5 for medium effects (a moderate difference between the groups); and 0.8 or more for large effects (a substantial difference between the groups) [18,19]. For one-way ANOVA analysis we obtained 0.84 power with a sample ES of 0.5 and an α error probability of 0.10. For paired *t*-test analysis we obtained 0.82 power with a sample ES of 0.6 and an α error probability of 0.10. For linear regression analysis we obtained 0.80 power with a sample ES value of 0.15 and an α error probability of 0.10 (Supplementary File S1). In a post hoc power analysis, a power value above 0.80 (or 80%) means that the statistical test had a high probability (≥80%) of detecting a true effect if one exists.

## 3. Results

### 3.1. Laboratory Values

Table 1 demonstrates the baseline characteristics of the participants. Body weight (kg) and BMI of patients in the obese group were higher than those in other groups (*p* < 0.001). The pH and HCO_3_ values of DKA patients were lower than the control, obese and post-DKA groups (*p* < 0.05). pH values approached normal levels following treatment in the DKA group (post-DKA group) and showed no significant difference compared to control and obese groups (*p* > 0.05). Although HCO_3_ values increased following treatment in the DKA group (post-DKA group), it was still lower than in the control and obese groups (*p* < 0.05). Urine ketone levels were higher in the DKA group than in the control, obese and post-DKA groups (*p* < 0.05). Urine ketone levels decreased significantly after treatment in patients in the DKA group (post-DKA group) and did not differ significantly compared to the control and obese groups. Blood glucose levels were higher in the DKA group than in the other groups (*p* < 0.05). Although blood glucose levels decreased significantly after treatment (post-DKA group), they were still higher than in the control and obese groups (*p* < 0.05). BUN and creatinine were higher in the DKA group vs. other groups (*p* < 0.05) and reached normal levels in the post-DKA group after treatment. GFR decreased in the DKA group vs. other groups (*p* < 0.05) and returned to normal levels in the post-DKA group after treatment. CRP values were higher in the obese, DKA and post-DKA groups compared to the control group (*p* < 0.05). HbA1c levels were higher in the DKA group than in the control and obese groups (*p* < 0.05). Triglyceride and very low-density lipoprotein (VLDL) cholesterol levels were higher in the DKA and obese groups vs. the control group (*p* < 0.05). The increase in triglyceride and VLDL was more pronounced in the DKA group. Albumin and Cl^−^ levels were lower in the DKA group vs. control and obese groups. In the obese group, fT4, ALT, insulin and HOMA-IR values were higher than in the control group (*p* < 0.05), while HDL cholesterol values were lower when compared to the control group.

### 3.2. Serum β-Hydroxybutyrate and Lactate Levels

**Figure 1:**
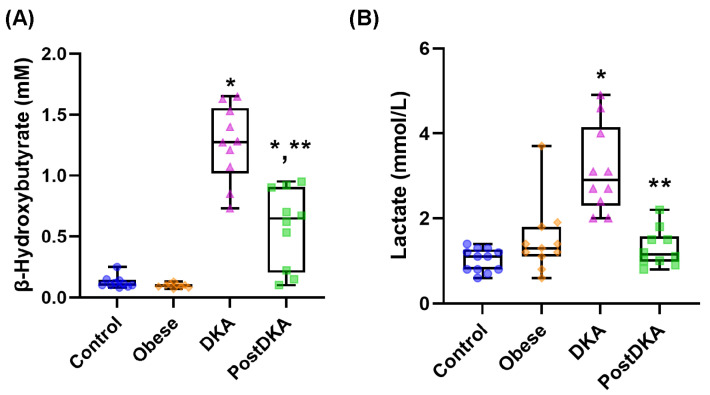
Box-plot graph of serum β-hydroxybutyrate and lactate levels. DKA, diabetic ketoacidosis. The 25th percentile is indicated by the box’s boundary that is closest to zero, the median is indicated by the line inside the box, and the 75th percentile is indicated by the box’s boundary that is furthest from zero. The 90th and 10th percentiles are indicated by whiskers above and below the box. Data represents results from 9–12 measurements. (**A**) Statistical analysis of β-hydroxybutyrate levels was performed using one-way ANOVA, with group differences assessed by Tukey’s multiple comparison test. Difference between DKA and post-DKA groups was determined by paired *t* test. *, *p* < 0.01, vs. control and obese groups. **, *p* < 0.01, vs. DKA. (**B**) Statistical analysis of lactate levels was performed using Kruskal-Wallis test, with group differences assessed by Dunn’s multiple comparison test. Difference between DKA and post-DKA groups was determined by paired *t* test. *, *p* < 0.01, vs. control and obese groups. **, *p* < 0.01, vs. DKA.

**Figure 2:**
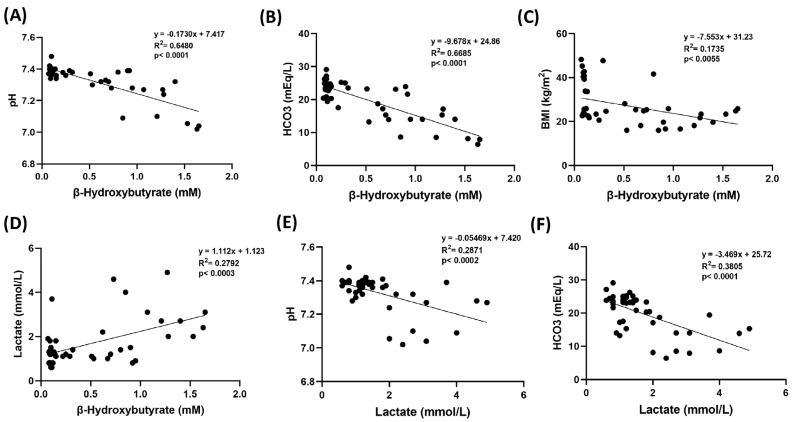
Correlation coefficients and scatter plots between (**A**) pH and β-hydroxybutyrate (**B**) HCO3 and β-hydroxybutyrate (**C**) BMI and β-hydroxybutyrate (**D**) Lactate and β-hydroxybutyrate (**E**) pH and lactate (**F**) HCO3 and lactate. Linear regression analysis was used to assess correlations. Statistical significance was established at a p-value of less than 0.01.

Serum β-OHB levels were measured in mM and reported as mean ± SD (Figure 1A). Measured β-OHB levels were higher in the DKA group (1.26 ± 0.31) compared to the control (0.12 ± 0.05), obese (0.10 ± 0.02) and post-DKA (0.58 ± 0.32) groups (*p* < 0.01). Although there was a significant decrease in β-OHB levels in the post-DKA group, it was still higher than in the control and obese groups (*p* < 0.01). Serum lactate levels were measured in mmol/L and reported as mean ± SD (Figure 1B). Lactate values were higher in the DKA group (3.15 ± 1.03) than in the control (1.03 ± 0.27), obese (1.49 ± 0.82) and post-DKA (1.30 ± 0.44) groups (*p* < 0.01). Lactate levels decreased in the post-DKA group and did not differ significantly from the control and obese groups. The pH and HCO_3_ values were significantly correlated with both β-OHB and lactate levels (*p* < 0.001) (Figure 2A,B,E,F). β-OHB levels were also correlated with both BMI (*p* < 0.01) (Figure 2C) and lactate levels (*p* < 0.001) (Figure 2D). Increased β-hydroxybutyrate and lactate levels were significantly higher in the DKA group and decreased with treatment. These metabolites strongly correlated with blood pH and HCO₃⁻ levels, reinforcing their utility as accessible markers of acidosis severity and metabolic derangement.

### 3.3. Serum TNF-α, IL-1β and N-SMase Values

**Figure 3:**
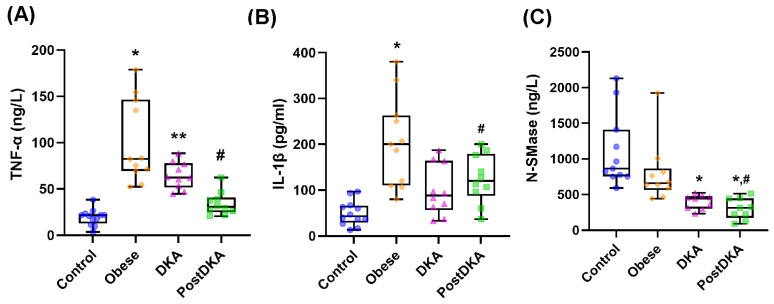
Box-plot graph of serum TNF-α, IL-1β and N-SMase levels. DKA, diabetic ketoacidosis. The 25th percentile is indicated by the box’s boundary that is closest to zero, the median is indicated by the line inside the box, and the 75th percentile is indicated by the box’s boundary that is furthest from zero. The 90th and 10th percentiles are indicated by whiskers above and below the box. (**A**) The data represent results from 10-12 independent experiments. Statistical analysis was performed using one-way ANOVA, with group differences assessed by Tukey’s multiple comparison test. Difference between DKA and post-DKA groups was determined by paired *t* test. *, *p* < 0.01, vs. control, DKA and post-DKA. **, *p* < 0.01 vs. control. #, *p* < 0.001 vs. DKA. (**B**) The data represent results from 10-12 independent experiments. Statistical analysis was performed using one-way ANOVA, with group differences assessed by Tukey’s multiple comparison test. Difference between DKA and post-DKA groups was determined by paired *t* test. *, *p* < 0.05, vs. control, DKA and post-DKA. #, *p* < 0.05 vs. DKA. (**C**) The data represent results from 9-11 independent experiments. Statistical analysis was performed using Kruskal Wallis test, with group differences assessed by Dunn’s multiple comparison test. *, *p* < 0.01, vs. control group. #, *p* < 0.05, vs. obese group.

Serum TNF-α levels were measured in ng/L and reported as mean ± SD (Figure 3A). Measured TNF-α values were higher in the obese group (100.20 ± 44.50) than in the control (19.46 ± 8.78), DKA (64.50 ± 14.32) and post-DKA (34.20 ± 11.62) groups (*p* < 0.01). TNF-α levels were higher in the DKA group than in the control group (*p* < 0.01) and decreased significantly after treatment (post-DKA group) (*p* < 0.001). Serum IL-1β levels were measured in pg/ml and reported as mean ± SD (Figure 3B). IL-1β values were higher in the obese group (203.94 ± 98.07) than in the control (49.72 ± 27.10), DKA (99.33 ± 54.77) and post-DKA (124.33 ± 53.36) groups (*p* < 0.05). IL-1β levels did not significantly differ in the control, DKA, and post-DKA groups. Serum N-SMase protein levels were measured in ng/L and reported as mean ± SD (Figure 3C). N-SMase values were lower in the DKA (395.22 ± 100.39) and post-DKA (300.78 ± 148.04) groups compared to the control (1105.73 ± 510.37) and obese (796.00 ± 428.76) groups (*p* < 0.05). Altered inflammatory cytokine profiles, with TNF-α and IL-1β levels elevated particularly in the obese group, and TNF-α showing significant reduction after DKA treatment. These changes highlight the interaction between inflammation and metabolic imbalance in DKA pathophysiology.

### 3.4. Serum Sphingolipid Levels

Serum sphingolipid levels are given in Table 2. The amounts of 16:0 SM, 18:0 SM and 24:0 SM were lower in the DKA and post-DKA groups than in the control and obese groups (*p* < 0.05). The measured serum sphingomyelins did not show a significant difference between the DKA and post-DKA groups. C18 CER, C20 CER, C22 CER and C24 CER levels were lower in the DKA and post-DKA groups than in the control and obese groups (*p* < 0.05). There was no statistically significant change in the long- and short-chain SM and CER ratios in the groups included in the study. The measured serum ceramides did not show a significant difference between the DKA and post-DKA groups. S1P amounts were lower in the DKA and post-DKA groups than in the control and obese groups (*p* < 0.05). The measured S1P levels did not show a significant difference between the DKA and post-DKA groups. Reduced serum sphingolipids (16:0–24:0 SMs and C18–C24 CERs) and decreased N-SMase activity in both DKA and post-DKA groups, suggests impaired sphingolipid metabolism. Given the role of sphingolipids in cell membrane integrity, signaling, and inflammation, these findings may indicate underlying mechanisms contributing to metabolic stress and tissue dysfunction in DKA.

**Figure 4:**
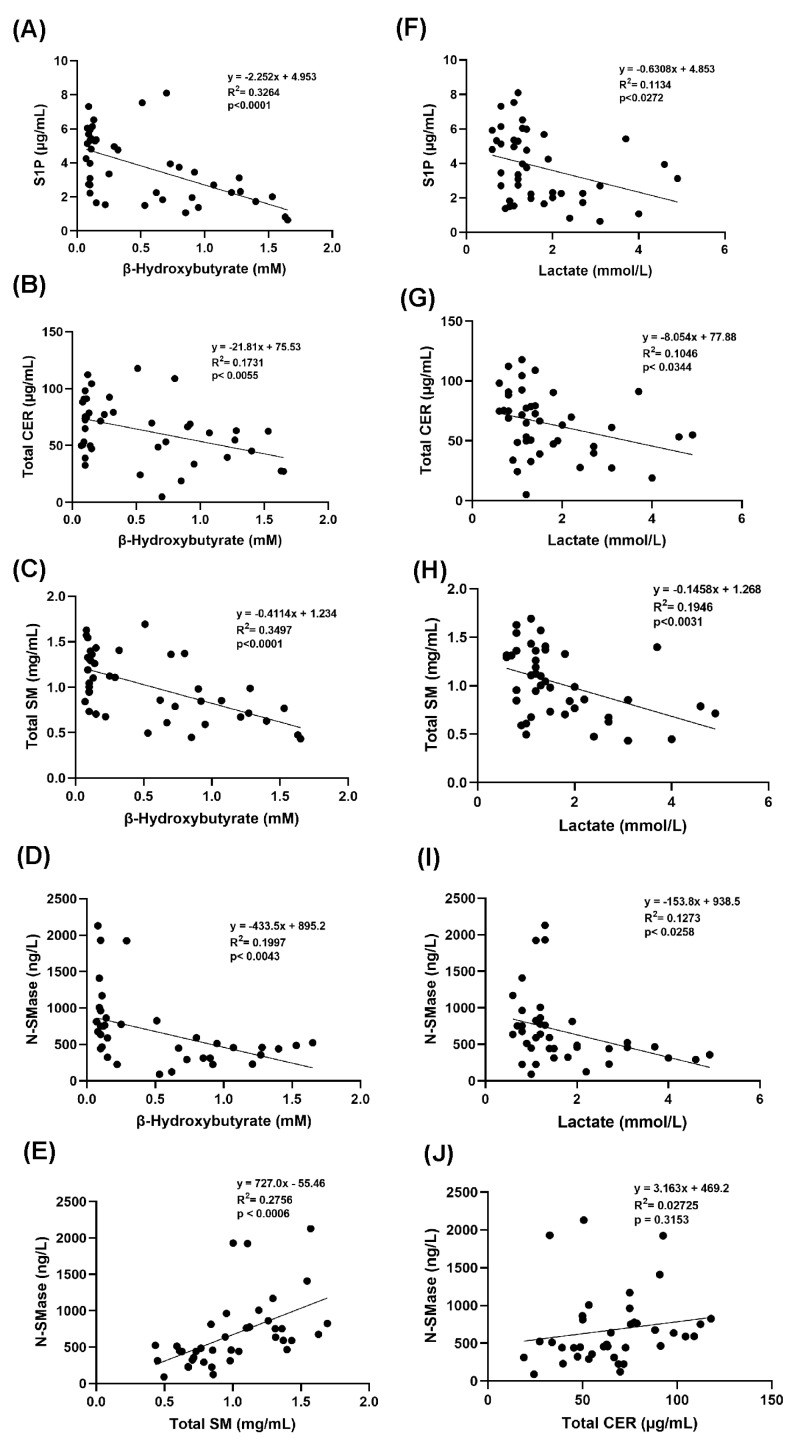
Correlation coefficients and scatter plots. (**A**–**D**) Linear regression analysis between S1P, total CER, total SM, N-SMase and β-hydroxybutyrate levels. (**E**) Linear regression analysis between N-SMase and total SM. (**F**–**I**) Linear regression analysis between S1P, total CER, total SM, N-SMase and lactate levels. (**J**) Linear regression analysis between N-SMase and total CER. Statistical significance was established at a *p*-value of less than 0.05.

Serum β-OHB and lactate levels were significantly correlated with S1P, total CER, total SM, and N-SMase values (*p* < 0.05) (Figure 4A–I). While the amount of serum N-SMase correlated significantly with total SM (*p* < 0.001), it did not correlate significantly with total CER (Figure 4C,E). Correlations among β-OHB, lactate, N-SMase, S1P, total SM, and total CER, supporting an integrated view of energy metabolism, lipid signaling, and inflammatory regulation.

## 4. Discussion

In this study, we observed significant metabolic and inflammatory alterations in individuals with DKA, including β-OHB and lactate levels, which decreased following standard DKA treatment. We also identified lower levels of N-SMase, sphingomyelins, and ceramides in the DKA and post-DKA groups compared to controls. Furthermore, correlations were found between β-OHB, lactate, and sphingolipid-related markers, suggesting a potential link between sphingolipid metabolism and DKA pathophysiology, marking the first sphingolipidomic study in this context. The data were compared to those from obese patients and healthy controls. Additionally, the levels of ceramides and pro-inflammatory cytokines were examined at both hospitalization and after treatment in DKA patients. The classification of participants into control (BMI ≤ 30, no health issues), obese (BMI > 30), and DKA (BMI ≤ 30) groups is grounded in established clinical guidelines and supported by previous studies. According to the World Health Organization (WHO), BMI categories are defined as follows: Normal weight: BMI 18.5–24.9; Overweight: BMI 25.0–29.9; Obesity: BMI ≥ 30.0 [20]. These classifications are widely accepted in clinical practice. All cases of ketoacidosis enrolled in our study had previously been diagnosed with type I diabetes, where patients often have lower BMI [21]. The control group served as a baseline to compare metabolic parameters and serum sphingolipid profile without the influence of obesity or DKA. The obese group was selected to investigate the impact of higher BMI on metabolic parameters and serum sphingolipid profile. The DKA group was selected to focus on DKA patients with BMI ≤ 30 to explore metabolic changes and sphingolipidomic profile in non-obese individuals experiencing DKA. This stratification allows for a clearer analysis of how obesity and DKA independently affect metabolic and sphingolipid parameters. By adhering to established BMI classifications and considering the documented prevalence of DKA in patients with BMI ≤ 30, this study aimed to provide insights into the metabolic distinctions among these groups.

The alterations in laboratory values observed in DKA patients are likely associated with hyperglycemia and increased lipolysis, which are known to play key roles in the metabolic disturbances seen in DKA. Hyperglycemia in DKA arises due to excessive glucose release from glycogenolysis and gluconeogenesis, combined with decreased glucose uptake in peripheral tissues, leading to glucosuria, osmotic diuresis, and sodium loss. This process causes dilutional hyponatremia, with serum sodium decreasing by 1.6 to 2.4 mmol/L for every 100 mg/dL increase in glucose above normal [11,12].

The second pathway involves lipolysis, which is triggered by elevated levels of stress hormones like epinephrine, cortisol, and glucagon in DKA. These hormones stimulate glycolysis and lipolysis, leading to increased lactate and free fatty acids in the bloodstream. The liver converts excess free fatty acids into triglycerides and VLDL, increasing serum triglyceride and VLDL levels [11,12]. Increased fatty acid oxidation also raises ketone production, resulting in metabolic acidosis, as indicated by decreased blood pH and bicarbonate levels [11,12]. Elevated inflammatory markers like CRP and TNF-α in DKA and obese patients [22] reflect the underlying inflammatory state, potentially contributing to insulin resistance. Stress hormones and ketone bodies, such as beta-hydroxybutyrate, further exacerbate the inflammatory response, activating immune cells to release TNF-α and increasing reactive oxygen species production, which can further disrupt insulin signaling pathways [23,24,25,26,27].

Chronic low-grade inflammation is a defining feature of obesity, and high levels of TNF-α and CRP are caused by several reasons, including immunological activation, metabolic abnormalities, and dysfunctional adipose tissue. Adipose tissue’s resident immune cells, including macrophages, and adipocytes both generate pro-inflammatory cytokines, such as TNF-α [28]. These cytokines, in turn, stimulate the liver to produce CRP, a marker of systemic inflammation, through activation of IL-6 [28]. TNF-α directly impairs insulin signalling by increasing serine phosphorylation of insulin receptor substrates (IRS), promoting insulin resistance [29]. Insulin resistance itself enhances lipolysis and FFA release, and elevated FFAs in obesity activate toll-like receptors (TLRs) on immune cells [24], triggering inflammatory pathways like NF-κB, which increases TNF-α production.

We have measured significantly lower amounts of SMs and CERs in DKA patients (Table 2). As indicated in the introduction, ketogenic diet has been shown to lower ceramide levels in humans [16] and β-OHB also causes a decrease in ceramides in rat muscle cells [17]. As previously mentioned, insulin insufficiency causes uncontrolled lipolysis in DKA, which releases a significant quantity of FFAs into the bloodstream. The liver may mainly use these FFAs for ketogenesis, which would limit their availability for the synthesis of ceramides and sphingolipids [30].

Ceramides are synthesized through pathways involving serine palmitoyltransferase (SPT) and subsequent steps that depend on the availability of fatty acid substrates. In DKA, the metabolic priority is shifted toward ketogenesis rather than complex lipid synthesis, potentially downregulating ceramide production [30]. The impact of insulin deficiency on the distribution of sphingolipids in muscle subcellular compartments were studied in C57BL/6 mice with streptozotocin-induced diabetes [31]. In the referenced study, FFA was elevated in both the total homogenates and sarcoplasmic fraction in insulin deficiency. After insulin therapy, plasma FFA levels returned to normal levels. Insulin deficiency had a significant effect on sphingolipid content and composition in the sarcoplasmic fraction, but less in the mitochondrial fraction [31].

De novo sphingomyelin biosynthesis begins by the condensation of serine and palmitoyl CoA via the action of SPT. The 18-carbon sphingosine base forms by 16 carbons from palmitic acid and 2 from serine. The addition of a fatty acid group leads to the formation of CER followed by synthesis of SM via subjoining of a phosphocholine headgroup [32]. The rate-limiting and regulating step in the de novo synthesis of ceramides appears to be catalyzed by SPT [32]. Serine palmitoyltransferase activity is positively correlated with tissue sphingolipid levels [33] and rises in hepatomas and liver regeneration when sphingolipid levels rise [34]. The pH profile of keratinocyte SPT activity revealed an alkaline pH maximum of 8.2 ± 0.4 [35] which was equivalent to that seen in rat liver microsomes [36]. The acidic environment in DKA may thus affect SPT enzymatic activity, involved in both CER and SM formation, potentially reducing their synthesis or promoting their degradation. Indeed, we observed that total SM and CER levels were significantly inversely correlated with β-OHB and lactate levels. Restoring normal metabolic conditions with insulin therapy and hydration helped reestablish CER and SM levels.

The direct result of sphingomyelin hydrolysis by sphingomyelinases is the production of ceramide and free phosphocholine [37]. The mammalian sphingomyelinases fall into three major categories based upon their pH optimum: acid sphingomyelinase, alkaline sphingomyelinase, and the N-SMase [37]. These three groups of enzymes have different subcellular distributions. Alkaline sphingomyelinase, expressed in the intestine and liver, plays a role in the digestion of dietary sphingomyelin [38]. The primary regulators of SM catabolism in most tissues are N-SMase and acid sphingomyelinase, which are widely expressed. N-SMase overexpression, causes degradation of sphingomyelin into ceramide [38]. We have observed decreased protein levels of N-SMase in DKA (Figure 3C) and found that protein levels of N-SMase were significantly positively correlated with total SM levels (Figure 4E). Currently, there is limited direct evidence linking increased total SM levels to upregulation of N-SMase protein levels. However, N-SMase activity can be influenced by regulatory factors such as oxidative stress, cytokines, and cellular stress responses [39]. Some studies suggest that substrate abundance like increased SM might influence enzymatic activity [39], but the relationship with protein expression levels of N-SMase is less straightforward and often depends on transcriptional and post-translational regulatory mechanisms [40]. It has been demonstrated that the chemotherapeutic drugs Daunorubicin and Camptothecin activate the promoter via certain transcription factors, hence stimulating N-SMase transcription in MCF-7 breast cancer cells and K562 leukemia cells. 147 base pairs upstream of exon 1 were found to represent the key promoter region needed for this activation. [41]. It was also shown that all-trans retinoic acid uses the similar method to cause N-SMase transcriptional activation [41].

We observed that sphingosine-1-phosphate (S1P) levels were decrease in DKA and that this decrease was significantly inversely correlated with β-OHB and lactate levels. The phosphorylation of sphingosine to S1P is catalyzed by sphingosine kinases (SphKs). Oxidative stress and decreased pH can suppress sphingosine kinase activity, reducing S1P production from sphingosine [42]. During DKA, cells prioritize ketone body production and fatty acid oxidation. This shift in lipid metabolism may also reduce the flux through sphingolipid biosynthesis pathways, indirectly affecting S1P levels. The sphingosine backbone irreversibly breaks down when S1P is cleaved by S1P lyase (SPL) [42]. Ceramide and S1P are kept in a dynamic balance within cells, which affects how the cells react to stress. By reducing the proliferative S1P signal, SPL can tip the scales in favor of cell death. Therefore, SPL is essential for preserving lipid homeostasis and proper cell fate responses [43].

The study included 11 females in the control group, 9 females in the obese group, and 5 females in the DKA group therefore it is important to acknowledge that cyclic insulin resistance during ovulation may influence metabolic parameters, including sphingolipid levels. Insulin resistance indices, such as HOMA-IR and adipose tissue insulin resistance index (ADIPO-IR) exhibit rhythmic cycling across the menstrual cycle [44]. However, this variation is significantly influenced by BMI, physical activity, and cardiorespiratory fitness [44]. These results suggest that individuals with high BMI or low fitness may be at greater risk of impaired insulin sensitivity, especially during the luteal phase [44]. Increased insulin resistance often results in elevated ceramide levels, which have been associated with impaired insulin signaling and metabolic dysfunction [4,5,6]. While direct evidence on the specific effects of cyclic insulin resistance on sphingolipids during ovulation is limited, the broader relationship between insulin resistance and ceramide levels suggests that these fluctuations could impact sphingolipid levels. However, given the acute nature of diabetic ketoacidosis and its profound metabolic disturbances, we anticipate that the effects of ovulation-related insulin resistance would be minimal in comparison. Additionally, due to the severity of DKA, the metabolic state is primarily driven by insulin deficiency and counterregulatory hormone activation, which is likely to overshadow any cyclic variations.

The study however also has several potential limitations. The study included only 33 participants, which were initially divided into three groups. The limited sample size may reduce the generalizability of the findings and affect the statistical power. The study focuses on acute DKA and post-DKA states but does not evaluate long-term changes in sphingolipid metabolism or cytokine levels, which might provide additional insights. Variations in underlying factors such as disease duration, glycemic control, and treatment regimens among participants could introduce variability. The study identifies correlations but does not investigate mechanisms linking sphingolipid metabolism, inflammation, and metabolic markers. The study excluded participants taking specific medications or supplements but did not account for other dietary or pharmacological factors that might impact sphingolipid metabolism. Addressing these limitations in future research would strengthen the findings and expand the understanding of DKA’s biochemical and therapeutic landscape.

## 5. Conclusions

**Figure 5:**
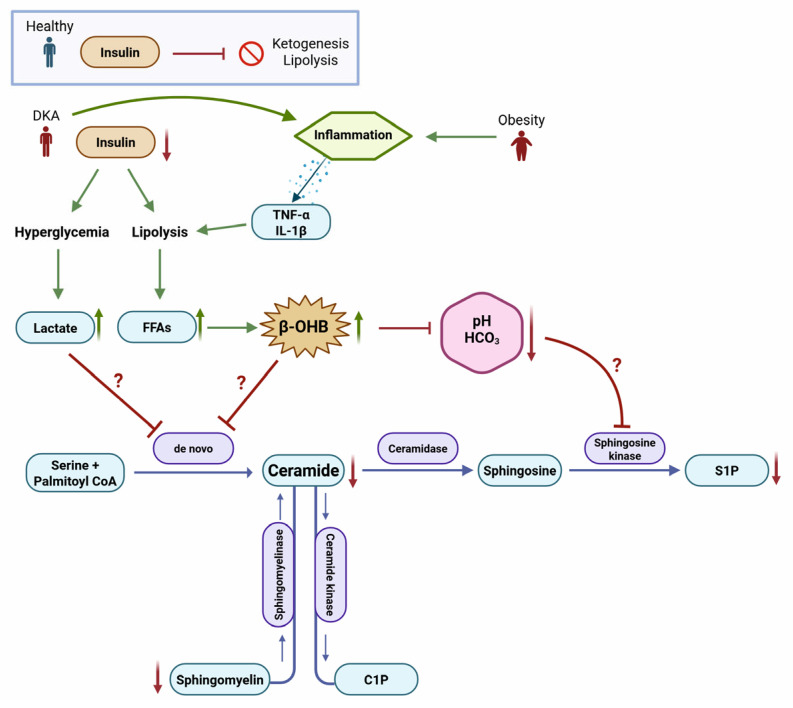
Key metabolic and inflammatory pathways affected in diabetic ketoacidosis (DKA) compared to healthy and obese states.

This study highlights the significant metabolic and inflammatory alterations observed in DKA and its resolution. Elevated levels of β-OHB, lactate, and pro-inflammatory cytokines, particularly TNF-α, were prominent during DKA, with partial normalization following treatment. The study also revealed reduced levels of sphingolipids, including SMs and CERs, as well as N-SMase, in DKA and post-DKA states. The observed correlations between metabolic markers, sphingolipids, and N-SMase provide insights into the complex biochemical interplay underlying DKA (Figure 5). These findings suggest potential pathways for therapeutic intervention, emphasizing the importance of targeting sphingolipid metabolism and inflammation in managing DKA and its associated complications. Collectively, these findings not only reinforce the diagnostic potential of routinely measured biomarkers (e.g., β-OHB, lactate, pH) but also uncover novel mechanistic insights into sphingolipid involvement in DKA. Identifying the persistent suppression of N-SMase and sphingolipids post-treatment suggests that biochemical normalization may lag behind clinical stabilization, a point which could inform future monitoring or therapeutic strategies.

## Figures and Tables

**Table 1:** Laboratory Values.

Parameter	Control (*n* = 12)	Obese (*n* = 11)	DKA (*n* = 10)	Post-DKA (*n* = 10)
Weight (kg)	64.28 ± 6.27	112.63 ± 17.96 ^a^	60.76 ± 11.90	60.76 ± 11.90
Height k (cm)	163.6 ± 7.6	164.73 ± 8.43	168.00 ± 11.87	168.00 ± 11.87
BMI (kg/m^2^)	23.97 ± 2.06	41.45 ± 4.77 ^b^	21.50 ± 3.65	21.50 ± 3.65
pH	7.39 ± 0.04	7.39 ± 0.01	7.17 ± 0.12 ^c, d^	7.34 ± 0.04
HCO_3_ (mEq/L)	24.71 ± 1.77	23.56 ± 2.19	11.38 ± 3.83 ^c, d^	18.25 ± 3.46 ^c^
Urine ketone (mmol/L)	0	0	2.60 ± 0.70 ^e, f^	0.56 ± 1.01
CR*P* (mg/L)	1.54 ± 1.92	14.22 ± 17.01 ^g^	40.39 ± 87.02 ^g^	37.29 ± 82.11 ^g^
Glucose (mg/dL)	77.67 ± 7.89	88.73 ± 9.96	446.50 ± 150.02 ^c, d^	182.50 ± 39.51 ^c^
BUN (mg/dL)	9.58 ± 2.84	11.50 ± 2.32	21.00 ± 11.48 ^g, d^	9.78 ± 3.23
Creatinine (mg/dL)	0.85 ± 0.14	0.80 ± 0.13	1.18 ± 0.35 ^e, d^	0.89 ± 0.23
GFR (ml/dk)	96.92 ± 13.65	104.27 ± 16.96	74.00 ± 29.43 ^h, f^	99.44 ±26.45
Na^+^ (mmol/L)	139.33 ± 1.72	139.45 ± 2.54	131.70 ± 3.71 ^c, d^	137.8 ± 6.5
K^+^ (mmol/L)	4.30 ± 0.22	4.37 ± 0.38	4.30 ± 0.85	3.95 ± 0.48
HbA1c (%)	5.33 ± 0.26	5.68 ± 0.35	11.29 ± 1.82 ^c^	-
TSH (mU/L)	2.14 ± 1.00	2.37 ± 1.43	2.26 ± 1.82	-
fT4 (ng/dL)	0.82 ± 0.10	1.20 ± 0.64 ^g^	0.92 ± 0.15	-
Total cholesterol (mg/dL)	196.17 ± 26.77	171.36 ± 34.20	214.50 ± 168.62	-
HDL-C (mg/dL)	64.58 ± 16.43	44.00 ± 9.49 ^g^	67.70 ± 62.71	-
LDL-C (mg/dL)	117.33 ± 28.30	102.90 ± 24.05	84.80 ± 45.97	-
VLDL-C (mg/dL)	14.25 ± 5.41	24.45 ± 12.86 ^g^	89.30 ± 206.63 ^g^	-
Triglyceride (mg/dL)	71.33 ± 26.61	121.91 ± 64.78 ^g^	446.30 ± 1033.94 ^g^	-
Cl^−^ (mmol/L)	103.50 ± 1.88	103.55 ± 1.92	99.50 ± 5.23 ^c^	-
Ca^2+^ (mg/dL)	9.41 ± 0.22	9.49 ± 0.48	9.09 ± 0.80	-
Albumin (mg/dL)	43.28 ± 3.61	42.23 ± 2.93	35.86 ± 6.90 ^e^	-
ALT (U/L)	13.67 ± 3.58	26.91 ± 11.82 ^g^	26.60 ± 27.79	-
AST (U/L)	16.25 ± 3.28	19.27 ± 4.56	19.33 ± 12.97	-
Lipase (U/L)	16.25 ± 6.81	24.45 ± 20.43	53.50 ± 97.22	-
Insulin (mIU/L)	6.69 ± 3.54	13.31 ± 5.40 ^i^	-	-
HOMA-IR	1.32 ± 0.82	3.03 ± 1.20 ^i^	-	-

All values are given as mean ± SD. BMI, body mass index; HCO_3_, bicarbonate; CRP, C-reactive protein; BUN, blood urea nitrogen; GFR, glomerular filtration rate; HbA1c, haemoglobin A1c; TSH, thyroid stimulating hormone; fT4, free thyroxine; HDL-C, high-density lipoprotein cholesterol; LDL-C, low-density lipoprotein cholesterol; VLDL-C, very low density lipoprotein cholesterol; ALT, alanine aminotransferase; AST, aspartate aminotransferase; HOMA-IR, homeostasis model assessment of insulin resistance; DKA, diabetic ketoacidosis. a, *p* < 0.001, vs. control, DKA and post-DKA. Statistical analysis was performed by Kruskal-Wallis one-way analysis of variance. The difference between the groups was determined by Dunn’s multiple comparison test. b, *p* < 0.001, vs. control, DKA and post-DKA. Statistical analysis was performed by one-way ANOVA. The difference between the groups was determined by Tukey’s multiple comparison test. c, *p* < 0.05, vs. control and obese group. Statistical analysis was performed by one-way ANOVA. The difference between the groups was determined by Tukey’s multiple comparison test. d, *p* < 0.05, vs. post-DKA. Statistical analysis was performed by paired *t* test. e, *p* < 0.05, vs. control and obese group. Statistical analysis was performed by Kruskal-Wallis one-way analysis of variance. The difference between the groups was determined by Dunn’s multiple comparison test. f, *p* < 0.01, vs. post-DKA. Statistical analysis was performed by Wilcoxon matched pairs signed rank test. g, *p* < 0.05, vs. control group. Statistical analysis was performed by Kruskal-Wallis one-way analysis of variance. The difference between the groups was determined by Dunn’s multiple comparison test. h, *p* < 0.05, vs. obese group. Statistical analysis was performed by Kruskal-Wallis one-way analysis of variance. The difference between the groups was determined by Dunn’s multiple comparison test. i, *p* < 0.01, vs. control group. Statistical analysis was performed by unpaired *t* test.

**Table 2:** Serum Sphingolipids Levels.

Parameter	Control (*n* = 12)	Obese (*n* = 11)	DKA (*n* = 10)	Post-DKA (*n* = 10)
Sphingomyelin (µmol/mL)				
16:0 SM (d18:1/16:0)	1.40 ± 0.23	1.24 ± 0.23	0.71 ± 0.19 ^a^	0.86 ± 0.36 ^b^
18:0 SM (d18:1/18:0)	0.13 ± 0.03	0.14 ± 0.05	0.05 ± 0.01 ^a^	0.06 ± 0.02 ^a^
24:0 SM (d18:1/24:0)	0.30 ± 0.08	0.28 ± 0.06	0.17 ± 0.06 ^c^	0.17 ± 0.07 ^c^
Ceramide (nmol/mL)				
C16 CER (d18:1/16:0)	3.97 ± 0.87	3.62 ± 0.42	2.92 ± 0.98	3.12 ± 0.59
C18 CER (d18:1/18:0)	1.03 ± 0.31	1.51 ± 0.59	0.71 ± 0.31 ^d^	0.76 ± 0.29 ^d^
C20 CER (d18:1/20:0)	1.79 ± 0.38	1.96 ± 0.94	0.80 ± 0.23 ^a^	0.96 ± 0.36 ^a^
C22 CER (d18:1/22:0)	26.20 ± 7.37	29.32 ± 7.47	16.18 ± 6.86 ^c^	15.81 ± 7.30 ^c^
C24 CER (d18:1/24:0)	89.65 ± 33.67	90.19 ± 21.55	50.64 ± 20.17 ^c^	53.85 ± 27.74 ^c^
S1P (nmol/mL)	13.74 ± 3.81	12.80 ± 3.28	5.43 ± 2.75 ^a^	6.82 ± 5.34 ^a^

All values are given as mean ± SD. SM, sphingomyelin; S1P, sphingosine-1-phosphate; CER, ceramide; DKA, diabetic ketoacidosis. a, *p* < 0.05, vs. control and obese groups. Statistical analysis was performed by Kruskal-Wallis one-way analysis of variance. The difference between the groups was determined by Dunn’s multiple comparison test. b, *p* < 0.05, vs. control group. Statistical analysis was performed by Kruskal-Wallis one-way analysis of variance. The difference between the groups was determined by Dunn’s multiple comparison test. c, *p* < 0.05, vs. control and obese groups. Statistical analysis was performed by one-way ANOVA. The difference between the groups was determined by Tukey’s multiple comparison test. d, *p* < 0.05, vs. obese group. Statistical analysis was performed by Kruskal-Wallis one-way analysis of variance. The difference between the groups was determined by Dunn’s multiple comparison test.

## Data Availability

Data obtained and analyzed in the work are not publicly available due to ethical limitations involving human subjects but are available from the corresponding author on reasonable request.

## Supplementary Materials

The following supporting information can be downloaded at: https://www.mdpi.com/article/10.3390/pathophysiology32030029/s1, Supplementary File S1: Post hoc power calculations; Table S1: LC-MS/MS validation parameters.

## Abbreviations

ALT	alanine aminotransferase
AST	aspartate aminotransferase
BMI	body mass index
CERs	ceramides
CRP	C-reactive protein
DKA	diabetic ketoacidosis
ECF	extracellular space
ELISA	enzyme-linked immunosorben
FFAs	free fatty acids
fT4	free thyroxine
GLUT-4	glucose transporter type 4
IL-1β	interleukin 1 beta
IL-6	interleukin 6
IRS	insulin receptor substrates
LC-MS/MS	tandem mass spectrometry
NF-κB	nuclear factor-kappa B
N-SMase	neutral sphingomyelinase
ROS	reactive oxygen species
SMs	sphingomyelins
SphKs	sphingosine kinases
SPL	S1P lyase
SPT	serine palmitoyltransferase
TLRs	toll-like receptors
TMB	tetramethylbenzidine
TNF-α	tumor necrosis factor alpha
TSH	thyroid stimulating hormone
VLDL	very low-density lipoprotein
β-OHB	body mass index
