# Supplementary material for: Diabetic Ketoacidosis Is Associated with Lower Serum Sphingolipids but Higher β-Hydroxybutyrate and Lactate: A Pilot Study
Source: Pathophysiology. 2025 Jun 26;32(3):29. doi: 10.3390/pathophysiology32030029 (PMC12285954; doi:10.3390/pathophysiology32030029)
Supplement: Supplementary file 1 [file pathophysiology-32-00029-s001.zip › Supplement Table 1.pdf]

Sphingolipid	Standards (ng/ml)	Intra-day CV (n=3)	Inter-day CV (n=3)	Accuracy (n=3)
**16:0 SM (d18:1/16:0)**	31.25	6,11%	11,62%	95,83%
	62.50	8,71%	9,53%	97,20%
	125	0,94%	2,54%	110,73%
	250	3,84%	2,14%	103,29%
	500	0,46%	1,24%	98,30%
**18:0 SM (d18:1/18:0)**	31.25	6,73%	5,58%	106,26%
	62.50	2,36%	5,86%	104,59%
	125	4,03%	1,06%	118,72%
	250	6,09%	3,73%	102,54%
	500	2,80%	2,77%	100,60%
**24:0 SM (d18:1/24:0)**	31.25	5,18%	9,92%	121,63%
	62.50	11,80%	10,51%	110,00%
	125	7,88%	3,68%	113,22%
	250	3,77%	7,18%	104,20%
	500	2,54%	0,71%	100,19%
**C16 CER (d18:1/16:0)**	31.25	5,81%	11,36%	101,60%
	62.50	7,46%	4,14%	108,80%
	125	6,51%	2,06%	112,43%
	250	1,81%	0,90%	100,23%
	500	4,86%	1,16%	100,24%
**C18 CER (d18:1/18:0)**	31.25	10,64%	3,58%	111,32%
	62.50	4,10%	5,31%	109,22%
	125	7,77%	3,45%	113,50%
	250	7,31%	10,23%	109,62%
	500	4,05%	1,70%	104,55%
**C20 CER (d18:1/20:0)**	31.25	5,99%	6,46%	103,46%
	62.50	6,74%	9,56%	99,53%
	125	3,81%	0,78%	100,51%
	250	5,37%	7,60%	108,18%
	500	2,78%	2,06%	96,75%
**C22 CER (d18:1/22:0)**	31.25	8,89%	12,23%	110,47%
	62.50	7,70%	1,75%	109,36%
	125	9,61%	2,85%	112,41%
	250	4,07%	2,99%	102,86%
	500	1,79%	3,61%	98,15%
**C24 CER (d18:1/24:0)**	31.25	14,40%	10,74%	111,59%
	62.50	4,28%	3,00%	101,66%
	125	1,70%	1,51%	102,55%
	250	3,47%	10,70%	109,73%
	500	2,77%	3,45%	93,42%
**S1P**	31.25	5,34%	5,12%	104,16%
	62.50	3,90%	4,82%	104,00%
	125	1,93%	3,18%	101,24%
	250	2,03%	6,46%	97,58%
	500	2,46%	6,67%	100,87%
