# Supplementary material for: Diabetic Ketoacidosis Is Associated with Lower Serum Sphingolipids but Higher β-Hydroxybutyrate and Lactate: A Pilot Study
Source: Pathophysiology. 2025 Jun 26;32(3):29. doi: 10.3390/pathophysiology32030029 (PMC12285954; doi:10.3390/pathophysiology32030029)
Supplement: Supplementary file 1 [file pathophysiology-32-00029-s001.zip › Supplement file 1.pdf]

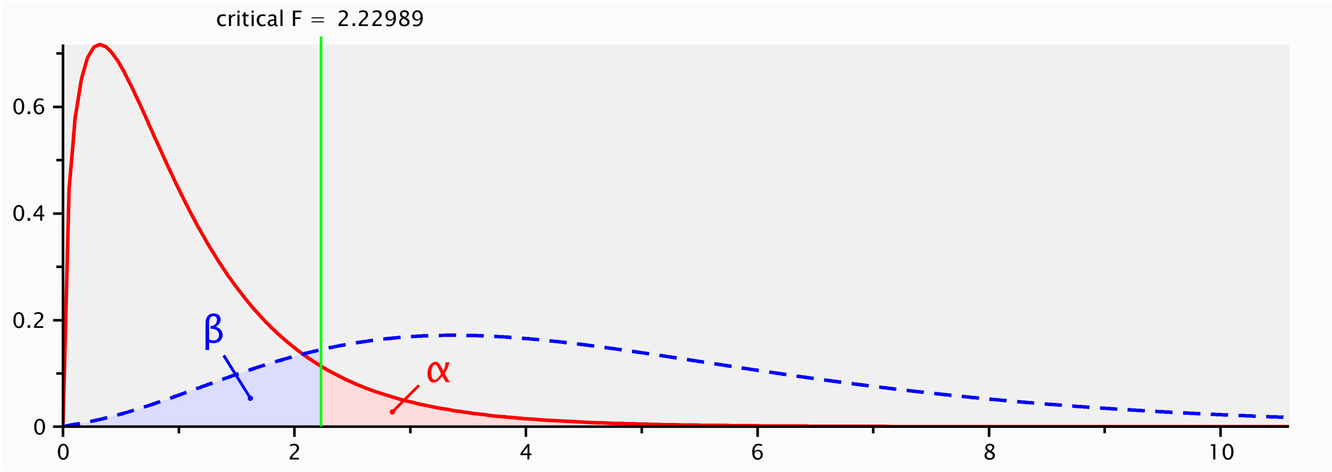

F tests - ANOVA: Fixed effects, omnibus, one-way
Analysis: Post hoc: Compute achieved power
Input: Effect size f = 0.50
α err prob = 0.10
Total sample size = 43
Number of groups = 4
Output: Noncentrality parameter λ = 10.7500000
Critical F = 2.2298896
Numerator df = 3
Denominator df = 39
Power (1-β err prob) = 0.8445472

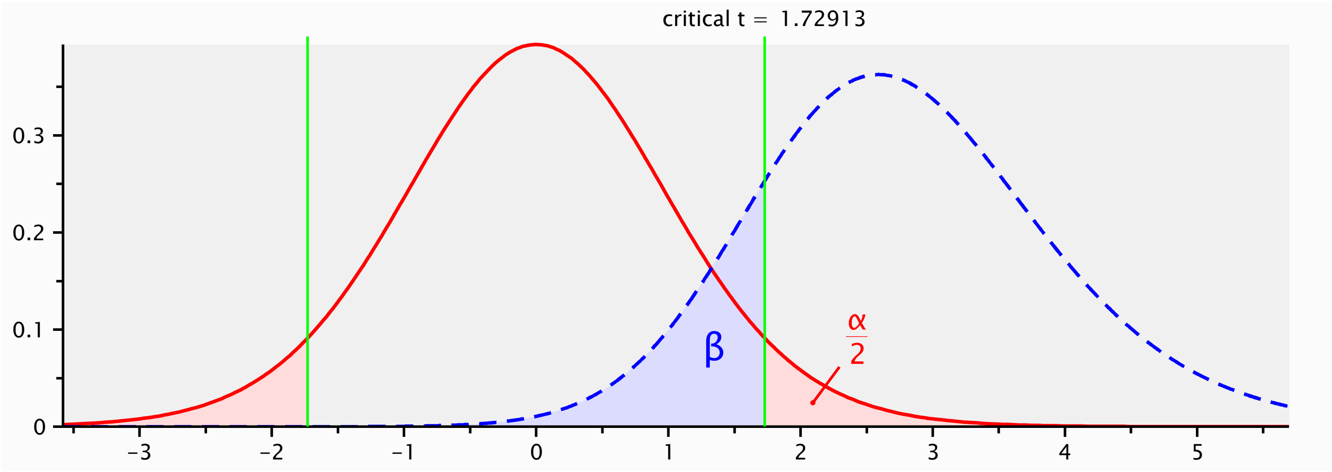

t tests - Means: Difference between two dependent means (matched pairs)
Analysis: Post hoc: Compute achieved power
Input: Tail(s) = Two
Effect size dz = 0.6
α err prob = 0.1
Total sample size = 20
Output: Noncentrality parameter δ = 2.6832816
Critical t = 1.7291328
Df = 19
Power (1-β err prob) = 0.8266508

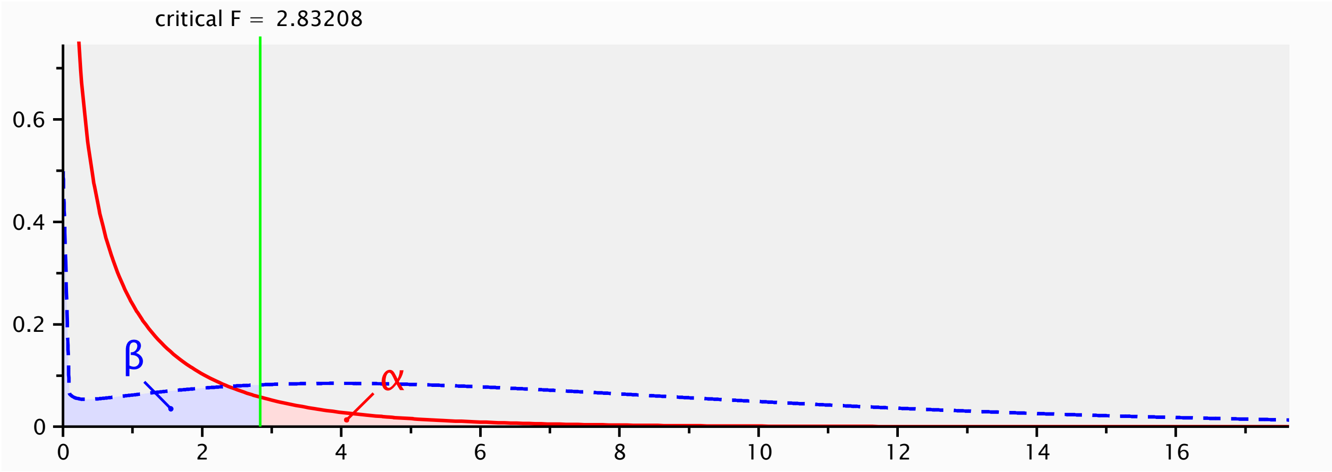

F tests - Linear multiple regression: Fixed model, R^2 deviation from zero
Analysis: Post hoc: Compute achieved power
Input: Effect size $f^2 = 0.15$
α err prob = 0.1
Total sample size = 43
Number of predictors = 1
Output: Noncentrality parameter λ = 6.4500000
Critical F = 2.8320784
Numerator df = 1
Denominator df = 41
Power (1-β err prob) = 0.8030652
